# Impaired mental rotation in benign paroxysmal positional vertigo and acute vestibular neuritis

**DOI:** 10.3389/fnhum.2013.00783

**Published:** 2013-11-26

**Authors:** Matteo Candidi, Alessandro Micarelli, Andrea Viziano, Salvatore M. Aglioti, Ilaria Minio-Paluello, Marco Alessandrini

**Affiliations:** ^1^Department of Psychology, “Sapienza” University of RomeRome, Italy; ^2^Istituto di Ricovero e Cura a Carattere Scientifico Fondazione Santa LuciaRome, Italy; ^3^Department of Clinical Sciences and Translational Medicine, “Tor Vergata” University of RomeRome, Italy

**Keywords:** vestibular neuritis, benign paroxysmal positional vertigo, vestibular disorder, mental imagery, body rotations, embodied cognition

## Abstract

Vestibular processing is fundamental to our sense of orientation in space which is a core aspect of the representation of the self. Vestibular information is processed in a large subcortical–cortical neural network. Tasks requiring mental rotations of human bodies in space are known to activate neural regions within this network suggesting that vestibular processing is involved in the control of mental rotation. We studied whether mental rotation is impaired in patients suffering from two different forms of unilateral vestibular disorders (vestibular neuritis – VN – and Benign Paroxysmal positional Vertigo – BPPV) with respect to healthy matched controls (C). We used two mental rotation tasks in which participants were required to: (i) mentally rotate their own body in space (egocentric rotation) thus using vestibular processing to a large extent and (ii) mentally rotate human figures (allocentric rotation) thus using own body representations to a smaller degree. Reaction times and accuracy of responses showed that VN and BPPV patients were impaired in both tasks with respect to C. Significantly, the pattern of results was similar in the three groups suggesting that patients were actually performing the mental rotation without using a different strategy from the control individuals. These results show that dysfunctional vestibular inflow impairs mental rotation of both own body and human figures suggesting that unilateral acute disorders of the peripheral vestibular input massively affect the cerebral processes underlying mental rotations.

## INTRODUCTION

Vestibular information is used to evaluate and maintain one’s own posture with respect to the vertical and to perceive the direction and velocity of one’s own head movement in space (Angelaki and Cullen, 2008). These functions are fundamental to our sense of self-location in space which is known to be further supported by the ability to integrate vestibular inputs with proprioceptive, motor, and visual information (Angelaki and Cullen, 2008; Cullen, 2012). Vestibular information is processed in a large neural network encompassing cortical [e.g., temporo-parietal-junction (TPJ), occipital, and frontal cortices) and subcortical (e.g., cerebellum) regions (Brandt and Dieterich, 1999; de Waele et al., 2001; Emri et al., 2003). Brain stimulation and imaging studies in healthy individuals and in neurologic patients suggest that TPJ plays a causal role in the integration of different types of information concerning our body position in space (Aglioti and Candidi, 2011; Lopez and Blanke, 2011) and thus supports a coherent sense of embodiment (Blanke et al., 2002, 2004, 2005; Lenggenhager et al., 2006; Ionta et al., 2011).

Alterations in vestibular inflow associated with peripheral vestibular failure disrupt the integrated representation of our body and movements in space and in fact in recent years it has been shown that neural activity within this cerebellar, subcortical, and temporo-parieto-frontal network is systematically changed early after the onset of vestibular deficits (Bense et al., 2004; Dieterich and Brandt, 2008, 2010; Alessandrini et al., 2013a).

Defective vestibular functions induce: (i) clear clinical symptoms such as a sudden onset of severe rotational vertigo associated with spontaneous nystagmus, nausea, vomiting, emotional disturbances, postural instability without any other neurologic or cochlear symptoms (Pollak et al., 2003; Strupp and Brandt, 2009); (ii) deficits in perceptual abilities concerning verticality and space representation (Bohmer and Mast, 1999a, b; Clement et al., 2009), motor behaviors (e.g., navigation, Peruch et al., 2005; Guidetti et al., 2008), cognitive functions (memory, attention, Smith et al., 2005; Hanes and McCollum, 2006); (iii) psychological disturbances (McKenna et al., 1991; Eagger et al., 1992; Gomez-Alvarez and Jáuregui-Renaud, 2011) and psychiatric symptoms such as depersonalization and derealization (Sang et al., 2006; Jauregui-Renaud et al., 2008; Gomez-Alvarez and Jáuregui-Renaud, 2011).

Research on mental rotations have identified at least two different kinds of mental transformations: *object-based mental transformation* (Shepard and Metzler, 1971) and *egocentric mental transformation* (Parsons, 1987). The basic idea is that object-based mental transformations are performed by reorienting mentally the object in order to solve a task, while egocentric mental rotations are solved by automatically imagining oneself rotating in order to assume the observed posture and solve the task. Object-based transformations have been studied by using joined blocks and non-tool objects although some of these stimuli might allow individuals to adopt non-rotational strategies (e.g., counting the blocks) allowing them to solve the task by judging object equivalence rather than performing a mental rotation. Previous literature suggests that subjects tend to use an object-based mental transformation (imagined rotation of the picture in space) when pictures of non-human objects are presented, but use an egocentric perspective-based mental transformation (imagined turning of oneself in space) for pictures of human bodies (Zacks and Tversky, 2005). One study by Lenggenhager et al. (2008), however, showed that this difference might not be stable by reporting that the strategy used by individuals did not depend on the visually presented stimuli (plant or human body in that study). Egocentric mental rotation might thus be better compared to object-based rotation by using the same stimulus (body images) but forcing individuals to use an “image-based” strategy in the latter condition. Mental motor imagery tasks (such as left/right judgments concerning an observed body) activate cortical regions largely overlapping with those activated during imagination and actual execution of actions (Parsons, 1987, 2003). Furthermore, imagining one’s own body rotations induces similar eye-movements to those associated to actual body rotation (Rodionov et al., 2004) and imagining oneself walking reduces spontaneous vestibular nystagmus to the same extent as in actual walking (Jahn et al., 2002). A consequence of the activation of these shared neural circuits during mental imagery rotations and actual body rotations is that perturbing activity in the peripheral vestibular system, i.e., alteration of the vestibular information inflow to cortical sites involved in higher-order processes, may disturb the performance of rotational mental imagery tasks. Indeed, according to the embodied theory of cognition (Barsalou, 2008), the same neural resources used to directly perceive a stimulus or perform a motor task are also called into causal play during the mental recreation (mental imagery) of that stimulus or sensorimotor state. Evidence for a causal role of sensorimotor correlates in the vicarious representation of seen and imagined sensorimotor states comes from behavioral and functional imaging studies on mental imagery (Kosslyn et al., 2000; Jeannerod, 2001; Ionta et al., 2007; Albright, 2012; Zvyagintsev et al., 2013) as well as from neuropsychological and brain stimulation studies (Fourkas et al., 2008; Moro et al., 2008; Bufalari et al., 2010; Avenanti et al., 2013).

In line with this view, experimentally induced vestibular stimulation, which is known to produce an imbalance in vestibular processing, does in fact affect the ability to perform mental imagery rotational tasks (Mast et al., 2006; Lenggenhager et al., 2008; Dilda et al., 2012). Lenggenhager et al. (2008) have shown that Galvanic Vestibular Stimulation (GVS) impairs the ability to perform left/right judgments about an observed human body image only if individuals mentally rotate their own body in space to match the orientation of the figure but not if they rotate the figure without imagining themselves rotating. Consistent with this, it has also been recently shown that actual body rotations influence the ability to perform own-body mental rotations in a direction-specific manner (van Elk and Blanke, 2013). The ability to mentally rotate one’s own body in space has been tested in studies on vestibular patients and individuals reporting dizziness (Grabherr et al., 2011; Wallwork et al., 2013).

In chronic vestibular patients (i.e., patients with vestibular loss), bilateral (but not unilateral) vestibular failure is known to induce the inability to perform mental imagery rotations of the whole body or body parts (Grabherr et al., 2011). A recent study showed that individuals experiencing dizziness do not show any difference between whole-body and body-part mental rotation (Wallwork et al., 2013) but do show a general impairment in mental rotation abilities suggesting that although rotational tasks may be more demanding for individuals suffering from dizziness, this effect might reflect a general inability to perform mental rotations regardless of the need to use one’s own body representation.

What is not known is whether the inability to perform mental rotations is specifically impaired in patients suffering from two forms of unilateral vestibular disorders such as acute vestibular neuritis (VN) and benign paroxysmal positional vertigo (BPPV) and whether this inability is specific to own-body rotations or expands to figure-based rotations as well.

Vestibular neuritis is a purely peripheral lesion of the vestibular system and constitutes an ideal “experimental model” of a partial vestibular de-afferentation as it offers the possibility of studying the effects of unilateral pathologically distorted vestibular inflow on mental imagery tasks. VN is one of the most common causes of vertigo, it is defined as a sudden, usually partial, unilateral failure of the peripheral vestibular organ without hearing impairments or any signs of brainstem dysfunction (Alessandrini et al., 2003, 2013a)).

Benign paroxysmal positional vertigo on the other hand is the most frequently observed pathology in otoneurological clinical practice. Patients experiencing this condition report acute vertigo when moving their head due to the presence of free otoconial debris migrating into a semicircular canal and resulting in abnormal and brief vestibular stimulation, accompanied by the prolonged loss of equilibrium (Blatt et al., 2000; Giacomini et al., 2002; Alessandrini et al., 2013b). It should be noted that, unlike VN, BPPV is not associated with vertigo when the head is kept stable.

Previous studies investigated extensively the nature of vestibular patients cognitive impairment (review in Smith et al., 2005; Hanes and McCollum, 2006) and have highlighted a spatial component. Based on previous studies investigating the ability to solve body-based and object-based rotations (Lenggenhager et al., 2008; Grabherr et al., 2011; Wallwork et al., 2013) in individuals and patients experiencing a vestibular dysfunction (either due to GVS, vestibular resection, or dizziness) we intended to compare the ability to solve mental rotations of human figures either using a “own-body” or an “object-based” strategy so to tap VN and BPPV visuo-spatial abilities. In this study we explored the ability of patients with acute peripheral vestibular disorders to perform two different visuo-spatial imagery tasks, namely “own-body” (egocentric) and “human-figure” (allocentric) rotation which are thought to require, respectively, high and low vestibular processing. In fact, egocentric rotations are thought to call into play vestibular processing to a greater extent than allocentric ones as they require the participant to imagine himself rotating in space (Parsons, 1987; Zacks and Tversky, 2005), possibly activating regions high up in the hierarchical organization of vestibular processing (TPJ; Blanke et al., 2005) and triggering vestibulo-ocular reflexes (Rodionov et al., 2004). Conversely, allocentric rotations are more strongly based on visuo-spatial manipulation, not necessarily calling into play vestibular processing (Shepard and Metzler, 1971), but tapping parietal functions (Zacks, 2008). Images of a human body were used in both conditions in order to keep the visual appearance of the stimulus identical in the two tasks. We tested two groups of patients suffering from different forms of unilateral vestibular disorders (VN and BPPV) and in a group of healthy controls (C). The aim was to detect the presence of any specific deficit in performing own-body and human-figure rotations in both the roll and yaw planes. Specifically, we tested (1) whether two different vestibular failures are reflected in a general or specific mental rotational impairment, and (2) whether “own-body” rotation is more seriously impaired than “figure-based” rotation. Any differential effect would speak in favor of a specific modulation of vestibular inflow on the activity of cortical and subcortical regions involved in the mental representation of one’s own orientation in space.

## MATERIALS AND METHODS

### PARTICIPANTS

All participants gave written consent to the protocol approved by the local ethic committee. The study was conducted in accordance with the Declaration of Helsinki.

#### BPPV Patients

Fourteen right-handed patients (eight female, six male; mean age 58 years) affected by a single and sole episode of BPPV caused by canalolithiasis of the right posterior semicircular canal (PSC) were enrolled in the study after a thorough otoneurological examination. Diagnosis of BPPV engaging the PSC was performed triggering a direction-changing torsional nystagmus by Dix–Hallpike maneuver (Dix and Hallpike, 1952; Bruno et al., 2007; Alessandrini et al., 2013b).

#### VN Patients

Nine right-handed patients (five female, four male; mean age 58 years) affected by a single and sole episode of right-sided VN were enrolled in the study after a neurological and otoneurological examination. The diagnosis was established according to the generally accepted criteria for this condition (Cooper, 1993; Strupp and Brandt, 2009; Alessandrini et al., 2013a): (1) sudden onset of vertigo and neurovegetative symptoms; (2) static and dynamic ataxia; (3) spontaneous, one-way, and persistent nystagmus with slow phase toward the affected ear detected by means of binocular electrooculography analysis; absence of (4) cochlear and (5) associated neurological symptoms or signs. T2-weighted and/or diffusion-weighted magnetic resonance imaging (MRI) sequences of the brainstem were acquired with a 1.5-T clinical MRI scanner to exclude the possibility that pseudoneuritis affected the vestibular nucleus or vestibular afferents within the brainstem.

Other exams were also conducted and consisted in caloric testing and subjective visual vertical. In the former case asymmetry was calculated by the formula of Jongkees from the slow-phase velocity, according to Honrubia (1994), vestibular paresis was defined as more than 25% asymmetry between the right-sided and the left-sided responses. In the latter case we used the binocular Bucket Test, according to Zwergal et al. (2009). All the patients showed unilateral vestibular paresis when studied by using caloric testing, the mean side asymmetry was 72%. With regard to the Bucket Test the mean of absolute deviation of subjective visual vertical was 8.6°.

***Exclusion criteria***.For both the BPPV and VN patients, exclusion criteria were based on the presence of major systemic illnesses, other vestibular disorders, psychiatric or neurological diseases, pregnancy, major vision illnesses, head trauma as well as the current and/or chronic use of medication.

#### Healthy individuals

Sixteen right-handed healthy volunteers (eight female, eight male; mean age 43 years) served as controls (C).

***Exclusion Criteria***.Exclusion criteria were based on the presence of major systemic illnesses, vestibular, psychiatric or neurological disorders, pregnancy, major vision illnesses, head trauma as well as the current and/or chronic use of medication.

### PATIENTS’ SETTING

All the participants performed the experimental procedures while seated in a noiseless and patient-friendly room. VN and BPPV patients performed the tasks within 3 and 10 days from the onset of symptoms and BPPV patients performed the tasks 30 min after the diagnostic Dix–Hallpike maneuver which insured they were not experiencing vertigo and nystagmus.

### TASKS

#### Task 1 “Own-Body Rotation”

**FIGURE 1:**
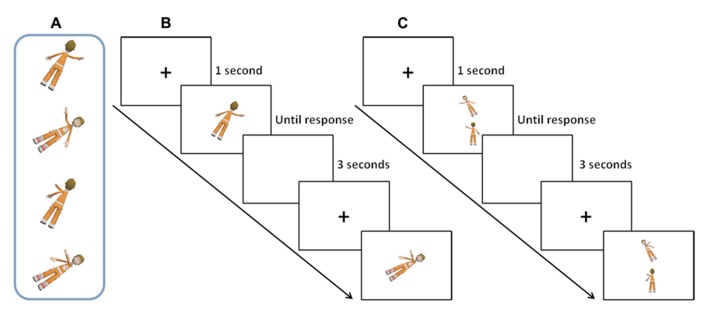
**Examples of stimuli and experimental trials from the two Tasks.** Panel **(A)** shows examples of the images used in the two Tasks. Panel **(B)** shows example trials through the timeline of the Task 1 figure (own-body rotation). Panel **(C)** shows example trials through the timeline of Task 2 figures (human-figure rotation).

Stimuli.Images depicting a human figure with one arm extended laterally and slightly raised or stretched laterally across the chest (i.e., crossing the vertical midline of the body). The vertical orientation of the figures was rotated through the roll plane by 30 or 60° with respect to the vertical in clockwise or anti-clockwise directions. Images of figures both facing the participant and with their backs to the participant were used (**Figure 1A**).

***Procedure***.In each trial a fixation cross was shown on the screen for 1 s before stimulus presentation. The body image appeared at the center of the screen and remained until the participant gave a response. Presentation of front view/back-view, 30°/60° rotated, left/right raised hand, and crossed/uncrossed arm was randomized within individuals. Individuals performed 48 trials in total in this Task (12 per each of the 4 conditions (30°-Back, 60°-Back, 30°-Front, 60°-Front).

***Task.***In this task participants were asked to mentally rotate their own body in order to reach the orientation and the posture of the stimulus. Once they had mentally performed this own-body rotation, they had to judge whether the right or left arm was raised in image of the human figure by pressing two keys on the keyboard (**Figure 1B**).

#### Task 2 “Human-Figure Rotation”

***Stimuli.***The same images as those used in Task 1 “Own body rotation” were used (**Figure 1A**). However, in this task two human figures were presented simultaneously on a screen, one in the upper part of the screen (Target) and one in the lower part (Sample). The Sample always represented the back-view of a human figure with either the left or right arm extended laterally and slightly raised or extended laterally across the chest (crossing the vertical midline of the body). The Target image represented a human figure in either a front- or back-view with the elevated hand that either matched (identical image) or did not match (different image) the Sample. The Target could be rotated with respect to the Sample image by either 30 or 60° so that the Task 2 figures matched the degree of rotation on the roll plane of Task 1 figure (i.e., when the Target was rotated 30° to the left, the Sample was up-right; when the Target was rotated 60° to the left, the Sample image was up-right; **Figure 1C**).

***Procedure***.At each trial a fixation cross appeared on the screen for 1 s before the Target and Sample images were presented. The pair of stimuli remained until the participant made a response. Presentations of back-to-back/back-to-front rotation, 30°/60° rotated, left/right raised hand and crossed/uncrossed arm were randomized within individuals. Individuals performed 48 trials in total in this Task (12 per each of the 4 conditions (30°-Back-Back, 60°-Back-Back, 30°-Back-Front, 60°-Back-Front).

***Task.***Individuals were asked to rotate the Sample image to the orientation of the Target and to judge whether the Sample image was identical to the Target by pressing two keys on the keyboard. In this task individuals were always asked to perform a back-to-front rotation when the Sample and the Target were presented in different views, thus matching the spatial rotation of the own-body task (**Figure 1C**).

The testing time for the rotational tasks was of about 30 min according to individual needs (i.e., familiarization with the tasks).

### DATA HANDLING

Individual mean Reaction Times (RTs) and Accuracy of response (ACC) were calculated for each experimental condition of the two tasks. Individuals that fell above or below 3 SD form group mean in a condition were discarded as outliers. Using this criterion we excluded 6 BPPV outliers (from an initial sample of 20 patients) no VN patients, and 4 controls (from an initial sample of 20 individuals). All RTs and ACC are provided in **Table 1**. RTs and ACC were log_10_-transformed in order to solve non-normality issues. Preliminary analyses were performed on individual RTs and ACC of response separately to test whether the experimental conditions showed any specific impact on each of these parameters. These two ANOVAs had Group as between factor and Angle (30°/60°), Rotation (Back-to-front/No-jaw rotation) and Task (Task1 figure/Task 2 figures) as within factors (results in **Table 2**). A correlation was run on RTs and ACC of each condition within each Group to test for any speed-accuracy trade off.

**Table 1:** The table reports the RTs and ACC of responses (mean ± standard deviation) for each group in all experimental conditions.

	30° Back		30° Face		60° Back		60° Face	
	RTs (ms)	ACC (%)	RTs (ms)	ACC (%)	RTs (ms)	ACC (%)	RTs (ms)	ACC (%)
**Task 1 own-body rotation (M ± SD)**
BPPV	2030 ± 732	99 ± 3	2251 ± 626	90 ± 13	2050 ± 794	96 ± 8	2364 ± 776	92 ± 1
VN	3169 ± 1592	92 ± 1	3600 ± 2415	81 ± 3	3587 ± 2184	90 ± 2	4202 ± 2509	80 ± 3
Controls	1274 ± 352	98 ± 6	1792 ± 800	93 ± 1	1356 ± 621	99 ± 2	1813 ± 663	95 ± 6
**Task 2 human-figure rotation (M ± SD)**
BPPV	3608 ± 1355	93 ± 9	4880 ± 2079	81 ± 1	4004 ± 1232	89 ± 1	4864 ± 2167	81 ± 1
VN	5412 ± 4452	80 ± 2	7677 ± 7235	68 ± 3	6255 ± 5743	74 ± 2	7469 ± 5085	64 ± 3
Controls	2486 ± 784	95 ± 6	3501 ± 1339	86 ± 2	2672 ± 933	94 ± 7	3745 ± 1828	87 ± 1

**Table 2:** The table reports the significant main effects of the ANOVAs performed on the log_10_Acc and log_10_RTs of responses.

	Effect	Significance
LogAcc	Group	*F*(2,36) = 7.323, *p* = 0.002
	Task	*F*(1,36) = 15.551, *p* < 0.001
	Rotation	*F*(1,36) = 13.959, *p* < 0.001
LogRTs	Group	*F*(2,36) = 11.893, *p* < 0.001
	Task	*F*(1,36) = 122.88, *p* < 0.001
	Angle	*F*(1,36) = 4.5949, *p* = 0.039
	Rotation	*F*(1,36) = 67.158, *p* < 0.001

*
No interaction resulted to be significant (all *p*s > 0.05). For a detailed description of *post hoc* comparisons please refer to the text.
*

In order to control for speed-accuracy trade off the main analysis of the study was performed on the index mean RTs/ACC for each individual’s condition. This decision is based on the advice of Salthouse and Hedden (2002) which suggest to analyze composite scores of RT and ACC after having tested the experimental effects on each of these parameter separately and after having tested whether RTs and ACC are correlated. The pilot analysis of RTs and ACC of responses showed that: (i) the same pattern of results was found in each of these parameters, and (ii) the two parameters resulted to be negatively correlated suggesting that the same effect was modulating RTs and ACC thus making it necessary to consider them in a combined manner (i.e., through the RTs/ACC index).

Thus in order to avoid speed-accuracy trade-off the main analysis was run on the ratio between RT and ACC in order to analyze participants’ behavior. Furthermore, RTs/ACC were log_10_-transformed in order to solve non-normality issues.

RTs/ACC indexes were processed with a mixed model repeated measures ANOVA with Group (BPPV/VN/Controls) as between factor, Task (1 figure/2 figures), Angle (30°/60°), and Rotation (No-jaw rotation/Back-to-Front rotation) as within subjects factors. The Bonferroni method was used to test *post hoc* of significant main effects and interactions.

After completing all the experimental procedures, individuals were asked to subjectively rate the degree to which they were able to actually use the “own body-rotation” and the “figure-rotation” strategy (i.e., “How much were you able to solve the task by imagining a rotation of your body?” and “How much were you able to solve the task by imagining a rotation of the sample image?”, respectively) during the two tasks by marking a 10 cm vertical Visual Analogue Scale (VAS) whose upper edge indicated a maximum value while the lower edge indicated a minimum value. A previous study reported a dissociation between the pattern of results found in individuals undergoing GVS while performing body mental rotations according to individuals’ egocentric and allocentric strategy for solving the task as measured via explicit ratings (Lenggenhager et al., 2008). Thus, in order to control for any possible between groups difference in the ability to use allocentric and egocentric strategies, we used these scales to exclude these might have influenced the results although it has to be recognized that subjective reports should be used cautiously.

## RESULTS

### STRATEGY

Data from one individual from the BPPV group, two individuals from the VN group and one from the Controls were discarded because of technical problems during Strategy recordings. Thus this analysis is performed on 35 individuals (13 BPPV, 7 VN and 15 Controls).

**FIGURE 2:**
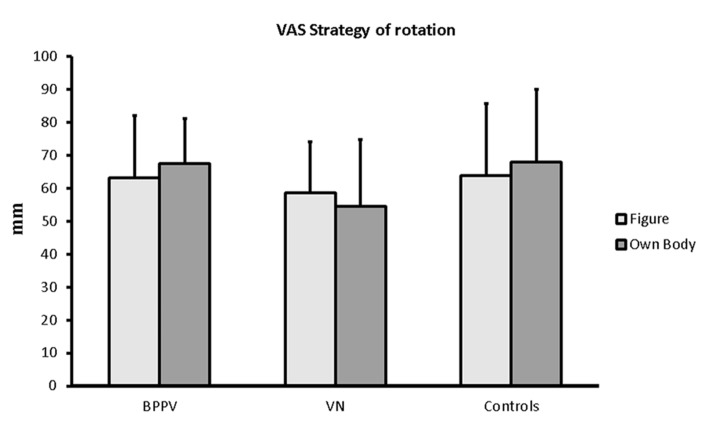
**Group VAS ratings (mean ± standard deviation) for the subjective evaluation of the ability to follow the strategies in the two tasks**.

Visual Analogue Scale ratings concerning the subjective evaluation of one’s own ability to follow the instructions were analyzed by means of a mixed model ANOVA with Group (BPPV, VN, and Control) as between group factor and Task (Own body and Figure) as within factor. None of the factors nor their interactions turned out to be significant (all *p* > 0.302) showing that the three groups of individuals were confident they were able to efficiently follow the instructions (**Figure 2**, mean ± SD).

### ROTATION TESTS

#### BPPV vs. VN vs. Controls

***Log_10_ACC.***Main effect of Group [*F*(2,36) = 7.323, *p* = 0.002] where VN (1.863 %) performed worse than BPPV (1.951%, *p* = 0.014) and controls (1.968%, *p* = 0.002) respectively. Main effect of Task [*F*(1,36) = 15.551, *p* < 0.001] with Test 1 figure being more easy than Task 2 figures. Main effect of Rotation [*F*(1,36) = 13.959, *p* < 0.001] where Back-to-front rotations were easier than No-jaw rotations (*p* = 0.001).

***Log_10_RTs.***Group main effect [*F*(2,36) = 11.893, *p* < 0.001] where controls (3.308) performed better than BPPV (3.462, *p* = 0.025) and VN (3.6078, *p* = 0.001) and BPPV tended to perform better than VN (*p* = 0.086).

Task 1 figure resulted to be easier than Task 2 figures [*F*(1,36) = 122.88, *p* < 0.001]. 60° roll rotations resulted to be more difficult than 30° ones [*F*(1,36) = 4.595, *p* = 0.039]. Back-to-front rotations resulted to be overall more difficult than No-jaw rotations [*F*(1,36) = 67.158, *p* < 0.001].

Furthermore, in order to control for any correlation between speed and ACC we run a correlation analysis on the two parameters. RTs and ACC of response resulted to be negatively correlated (*p* < 0.05) in 6 out of 8 (2 Tasks × 2 Rotations × 2 Angles) conditions indicating slower RTs were associated to lower ACC across all groups. Although the negative correlation speaks against any speed-accuracy trade off, the two parameters were combined together and the RTs/ACC index was analyzed.

***Log_10_(RTs/ACC).***The mixed model repeated measures ANOVA on the Log_10_-transformed RTs/ACC indexes highlighted a significant main effect of Group [*F*(2,36) = 15.358, *p* < 0.001]. The VN group was more seriously impaired in solving the tasks with respect to both BPPV (*p* = 0.011) and Controls (*p* < 0.001). BPPV group was more impaired than Controls (*p* = 0.035).

The Task also reached statistical significance [*F*(1,36) = 112.57, *p* < 0.001] with Task 2 figures being more difficult than the Task 1 figure.

Performance was more difficult during 60° rotations on the roll plane in all groups as indicated by the significant main effect of Angle [*F*(1,36) = 7.560, *p* = 0.009].

As expected, the Back-to-front rotations were significantly more difficult than the No-jaw rotation condition in both Tasks [main effect of Rotation, *F*(1,36) = 91.606, *p* < 0.001].

Back-to-front rotations however were comparatively more difficult with respect to No-jaw rotations in the Task 2 figures than during the Task 1 figure as suggested by the significant Task × Rotation interaction [*F*(1,36) = 4.253, *p* = 0.046]. *Post hoc* comparisons indicated that Back-to-front rotations were more difficult than No-jaw rotations in the Task 1 figure (*p* < 0.001) and that this difference was even greater in Test 2 figures (*p* < 0.001).

No other interaction reached statistical significance (all *p*s > 0.193 for the interaction Angle × Group).

Crucially no interaction with the Group factor reached statistical significance (all *p*s > 0.193 for the interaction Angle × Group) indicating that the three groups of individuals were not differently affected by any task and experimental condition.

**FIGURE 3:**
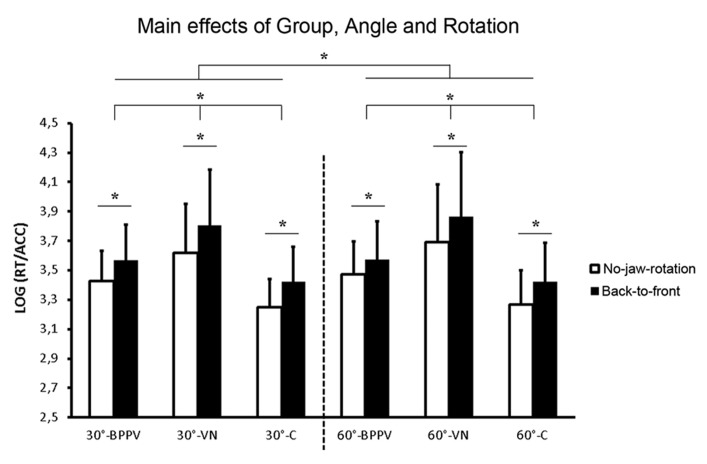
The main effect of Group averaged between Task 1 and 2 figures and shown in all experimental conditions (30°/60°, Back-to-front/No-jaw rotations; mean ± standard deviation). *p < 0.05.

All experimental conditions for the three groups averaged across Task 1 and 2 figures are shown in **Figure 3**.

## DISCUSSION

The main result of the present study is that VN and BPPV patients are more impaired than controls in performing mental rotation tasks although these two pathological conditions differ for a number of clinical symptoms. In fact, while VN is associated with nausea, nystagmus and intense vertigo in its acute phase, no such symptoms occur in BPPV when patients stay still in an upright seated posture (which is the condition adopted in our experimental tasks). Thus, the different forms of peripheral vestibular dysfunction cause mental rotation deficits regardless of the specific clinical condition. We propose that finding similar pattern of results in the Test 1 and 2 figures suggests that patients were more challenged by spatial rotations in general, regardless of the vestibular involvement, and that a more demanding task (i.e., Task 2 figures) might have tapped individuals spatial abilities to a greater extent but failed to highlight any specific interaction with the Groups, thus suggesting that patients impairments regarded mental rotation in general and not spatial processing *per se.*

### BPPV

The BPPV pathophysiology is believed to be linked to the presence of free otoconial debris migrating into semicircular canals of the inner ear during head movements, resulting in an abnormal stimulation of the ampullary crests (Brandt and Steddin, 1993). This phenomenon is thought to be the cause of the acute rotatory vertigo experienced by patients when they move their head. However, after the acute attacks associated with head movements, BPPV patients often also report prolonged problems with balance of unclear origin (Vicini and Campanini, 1995) which can last for weeks and subsides spontaneously regardless of the therapy followed. This prolonged dizziness has been more difficult to study (Di Girolamo et al., 1998) but has been characterized by using static posturography approaches which show a postural imbalance on the anterior-posterior plane 3 days after treatment with the Epley maneuver (Giacomini et al., 2002).

Previous studies have shown that peripheral vestibular deficits are associated with cognitive impairments under conditions of postural challenge which is clearly critical for vestibular patients (Yardley et al., 2001; Smith et al., 2005; Hanes and McCollum, 2006). Significantly, it has to be noticed that well compensated patients suffering from benign paroxysmal positional nystagmus show mild cognitive impairments in specific tasks although they tend to perform normally in the Digit Span test (which tests memory span by measuring the length of a sequence of random numerals that can be repeated by the subject) and the Mini Mental State Exam (which tests orientation, attention, immediate and short-term recall, language, and ability to comprehend and follow commands; Erickson et al., unpublished). Similarly Yardley et al. (1998) showed that individuals experiencing mild forms of dizziness demonstrate a connection between mild balance/vestibular problems and persistent cognitive difficulties.

Here we show for the first time that mental rotational tasks are able to capture BPPV impairments in cognitive tasks which may represent a consequence of their mild postural challenges. More importantly we show that mental rotation tasks permit the tracking of unilateral vestibular dysfunctions in two clinical conditions characterized by profound differences in the pattern of symptoms. This suggests that altered central vestibular processing may be the common cause of the rotational impairments in these patients rather than the symptoms which are different in the two pathologies. In view of this, the present findings do not exclude a causal role of vestibular disfunctions in performing mental rotation tasks (embodied cognition hypothesis) and in fact they strengthen the idea that altered vestibular inflow may result in graded impairments in spatial cognition.

### VESTIBULAR NEURITIS

The fact that VN patients performed worse overall is unlikely to depend on any pathological ocular behavior (nystagmus) since although Test 2 figures is visually more demanding than the Test 1 figure, the impairment in the two tests in VN patients follows the same pattern with respect to BPPV and Controls. As the angle and the rotation also made the tasks incrementally difficult for VN patients, the indication is that these patients were impaired in mentally rotating their own body as well as a visual stimulus. The absence of interaction between the Group and the other factors that reached statistical significance as main effects (Angle, Task, and Rotation) shows that VN patients were affected by these factors to the same extent as the BPPV group and the Controls. Furthermore, the significant Task by Rotation interaction shows that VN patients followed the same behavioral pattern as BPPV patients and Controls when performing the tasks.

For the same reason, it seems unlikely that the known reduction in activity in visual cortices which is found at early stages from VN onset (Bense et al., 2004; Dieterich and Brandt, 2008; Alessandrini et al., 2013a) and during vestibular stimulation (Bense et al., 2001; Brandt et al., 2002), and which is considered to be a form of neural compensation in order to reduce the visual processing of unstable information due to nystagmus, is the cause of the pattern of results. The amount of mental rotation to solve the tasks is the critical factor that impairs VN performance in both visually less demanding tasks (Test 1 figure) and in a more visually demanding task (Test 2 figures) with respect to the others individuals. Furthermore, we wish to emphasize here that while it is possible that a reduction in metabolic activity in visual areas may explain the impairment in the VN group, this does not hold true for the BPPV group.

These functional changes are also reflected in morphological alterations in VN patients (Helmchen et al., 2009; zu Eulenburg et al., 2010). A voxel-based morphometry study on VN patients 2.5 years after onset showed changes in the vestibular system (increases in gray matter in medial vestibular nuclei and in white matter in the pontine commissural vestibular fibers), somatosensory system (increased gray matter in the right gracile nucleus) and visual motion-related areas in those patients with residual vestibular hypofunction (increased gray matter in MT/V5; zu Eulenburg et al., 2010).

As suggested by previous studies on labyrinthectomy patients and individuals experiencing dizziness (Grabherr et al., 2011; Wallwork et al., 2013), the fact that VN and BPPV patients performed worse in the rotation of a human figure with respect to their performance when they were directly asked to mentally rotate themselves in space shows that vestibular deficits may not specifically affect the ability to perform own-body based mental rotations but rather they affect the ability to mentally rotate a seen stimulus regardless of the strategy.

In similar tasks to the one we used here, Grabherr et al. (2011) used an own-body (egocentric based, in their words) and a figure (object based, in their words) task and found similar impairments in the two tasks in bilateral but not unilateral vestibular patients. Furthermore, the fact that we observe impairments in unilateral VN patients while they fail to report this might be due to differences between the pathologies under study. Indeed, while Grabherr et al. included in the unilateral group individuals who underwent labyrinthectomy (mainly due to Meniere’s disease) 8 years before testing, we tested unilateral patients in their acute phase. The study by Grabherr et al. (2011) on unilateral and bilateral vestibular resected patients failed to find any rotational impairments in unilateral patients supporting the idea of a “graded impact” of vestibular processing in solving mental rotational issues (i.e., when correct vestibular inflow is maintained by one ear the rotations are made possible, not when both vestibular nerves are resected). Further studies are needed in order to better qualify the impact of unilateral and bilateral vestibular processing to solve mental rotation tasks.

Significantly, VN patients showed that they were overall more impaired on both the roll and the yaw plane suggesting that the disease impacts both rotational planes equally. If this were the case, the impaired ability to rotate human figures on the roll plane using an “egocentric” strategy found in healthy individuals undergoing GVS (Lenggenhager et al., 2008) might indicate a different relationship between experimentally induced vestibular stimulation, vestibular deficits and mental imagery abilities.

Both unilateral vestibular stimulation and unilateral failure of the vestibular endorgan create a vestibular tonic imbalance; however, this imbalance occurs at different levels of vestibular system activity (i.e., the trigger zone at the hair-cell afferent axon interface and the pars medialis of the utricular macula for GVS, vestibular nerve in VN and macula for BPPV; Lobel et al., 1998; Bense et al., 2001; Fitzpatrick and Day, 2004; Teggi et al., 2013). A similar issue was discussed by Bense et al. (2004), who showed that VN is associated to a pattern of tonic cerebral metabolic changes which is not identical to that following vestibular stimulation through caloric testing and GVS (Bense et al., 2001; Lobel et al., 1998). Beside a similar activation-deactivation in vestibular-visual cortices in patients with vestibular failure (VN) and healthy volunteers during experimental vestibular stimulation, these authors reported a difference at the cortical level where VN patients only show a contralateral increase of metabolism in PIVC which they ascribe to either (1) a depression of the more dominant ipsilateral right-sided ascending projections to the right insular cortex by right-sided VN, because there is no tonic endorgan input (resting discharge), or (2) a left-sided vestibular excitation due to a higher resting discharge rate of the unaffected left vestibular nuclei complex induced by the acute right-sided vestibular failure. With respect to the results of the present paper and the comparison between similar task execution in healthy individuals undergoing GVS, it has to be noticed that Lenggenhager et al. (2008) study only asked participants undergoing GVS to perform rotations on the roll plane since GVS is known to predominantly evoke illusory motion of both body and visual field in the roll plane (due to the response of semicircular canal organs and the medial part of the utricular macula; Fitzpatrick and Day, 2004). These differences, and their interactions with cognitive strategies, may explain the different pattern of results found in the ability to solve mental rotation tasks found in healthy individuals undergoing GVS (Lenggenhager et al., 2008) or actual body rotations (van Elk and Blanke, 2013).

### MENTAL ROTATIONS AND GRAVITATIONAL PROCESSING

The relation between gravitational information and mental rotation tasks have been tackled by studies in conditions of altered gravity (microgravity and increased gravity) in cosmonauts (Matsakis et al., 1993; Leone et al., 1995; Grabherr et al., 2007; Dalecki et al., 2013). Studies comprising object rotation in altered gravity setting reported inconsistent result (either a facilitation (Matsakis et al., 1993) or no effect (Leone et al., 1995) on rotational abilities in microgravity). Grabherr et al. (2007) studied mental rotation in microgravity specifically with respect to body and body parts rotations. These authors report higher error rates for both bodies and body parts in microgravity with respect to 1 g and no such effect for object rotations. Furthermore, RTs showed a congruent pattern whith longer RTs in microgravity with respect to 1 g (and even longer for body parts than full body rotations). In the object rotation task no such effect on RTs were observed. The results supported the view that vestibular information are crucial in a spatial embodiment framework which holds that imagined body transformation is likely to use some gravitational reference information regarding the actual body position whereas this is not necessary for object-based mental rotations. A missing update about body orientation with respect to gravity (a condition that may be experienced in VN and BPPV and microgravity) could thus interfere with the task of mentally transforming one’s own body. During exposure to microgravity, vestibular input from the otoliths about the direction of gravity is absent (apart from the resting discharge level) while during VN and BPPV there is an unbalance in bilateral vestibular information which triggers vertigo and balance problems. As discussed, several cortical sites receive vestibular input, and a missing update about the direction of gravity, or a corrupted inflow, could interfere with tasks in which this information is involved.

## CONCLUSION

The results of the present study showed that mental rotation of both own-body and human figures (egocentric and allocentric, respectively) were impaired in acute vestibular patients with respect to healthy controls and especially so in individuals suffering from VN with respect to other forms of vestibular disorder (BPPV). The present findings expand previous literature (Yardley et al., 2001; Smith et al., 2005; Hanes and McCollum, 2006; Grabherr et al., 2011; Wallwork et al., 2013) by showing that vestibular patients in two different neurological and clinical conditions (namely VN and BPPV) show difficulties in performing mental rotations regardless the use of egocentric and allocentric strategies but that spatially demanding tasks may more strongly tap their visuo-spatial difficulties. This evidence is in line with the notion that individuals experiencing vestibular dysfunction do not show any difference between whole body and human figure mental rotation and supports the idea that vestibular failures are reflected in a general inability to perform mental rotations regardless of the need to use one’s own body representation and regardless of the specific clinical condition.

## Conflict of Interest Statement

The authors declare that the research was conducted in the absence of any commercial or financial relationships that could be construed as a potential conflict of interest.

## AUTHOR CONTRIBUTIONS

Matteo Candidi, Alessandro Micarelli, Ilaria Minio-Paluello, Salvatore M. Aglioti, Marco Alessandrini designed the study; Alessandro Micarelli, Andrea Viziano, Marco Alessandrini performed the study; Matteo Candidi, Alessandro Micarelli, Marco Alessandrini analyzed data; Matteo Candidi, Alessandro Micarelli, Salvatore M. Aglioti, Marco Alessandrini wrote the paper.
